# Molecular mechanisms in colitis-associated colorectal cancer

**DOI:** 10.1038/s41389-023-00492-0

**Published:** 2023-10-26

**Authors:** Royce W. Zhou, Noam Harpaz, Steven H. Itzkowitz, Ramon E. Parsons

**Affiliations:** 1 The Tisch Cancer Institute, Icahn School of Medicine at Mount Sinai, New York, NY USA; 2 Department of Oncological Sciences, Icahn School of Medicine at Mount Sinai, New York, NY USA; 3 Molecular Medicine Program, Internal Medicine Residency Program, Department of Medicine, University of California San Francisco, San Francisco, CA USA; 4 The Dr. Henry D. Janowitz Division of Gastroenterology, Icahn School of Medicine at Mount Sinai, New York, NY USA; 5 Department of Pathology, Icahn School of Medicine at Mount Sinai, New York, NY USA

**Keywords:** Cancer genetics, Cancer genomics

## Abstract

Sustained chronic inflammation of the large intestine leads to tissue damage and repair, which is associated with an increased incidence of colitis-associated colorectal cancer (CAC). The genetic makeup of CAC is somewhat similar to sporadic colorectal carcinoma (sCRC), but there are differences in the sequence and timing of alterations in the carcinogenesis process. Several models have been developed to explain the development of CAC, particularly the “field cancerization” model, which proposes that chronic inflammation accelerates mutagenesis and selects for the clonal expansion of phenotypically normal, pro-tumorigenic cells. In contrast, the “Big Bang” model posits that tumorigenic clones with multiple driver gene mutations emerge spontaneously. The details of CAC tumorigenesis—and how they differ from sCRC—are not yet fully understood. In this Review, we discuss recent genetic, epigenetic, and environmental findings related to CAC pathogenesis in the past five years, with a focus on unbiased, high-resolution genetic profiling of non-dysplastic field cancerization in the context of inflammatory bowel disease (IBD).

## Clinical perspective of CAC

The primary driver of colorectal carcinogenesis in IBD is chronic inflammation. The two main types of IBD, Crohn’s disease and ulcerative colitis, typically begin in young adulthood and persist throughout life [1]. Epidemiological and clinicopathological data link colonic inflammation with the development of CAC: the risk of CAC rises steadily with the duration and extent of colonic inflammation, whereas there is no increased risk of CAC in patients with IBD limited to the rectum (proctitis) with no appreciable colonic inflammation [2]. Furthermore, a meta-analysis of 40 studies finds extensive ulcerative colitis to be associated with increased risk of advanced colorectal neoplasia compared to just left-sided ulcerative colitis (odds ratio: 2.43) [3].

The chronically inflamed colon can develop anatomic abnormalities such as inflammatory polyps (so-called “pseudopolyps”), strictures, and eventually shortening of the colon. Although such changes have historically been considered risk factors for developing CRC, more recent analyses indicate that they may not independently predict CAC beyond the chronic inflammation that gave rise to them in the first place. One of the strongest risk factors for CAC is primary sclerosing cholangitis (PSC), a chronic inflammatory condition of the bile ducts that is found in up to 10% of patients with IBD. Individuals with IBD and concomitant PSC have a 5-9-fold increased risk of CAC compared to IBD patients without PSC [4]. Ironically, in this clinical setting, the colonic inflammation is typically very mild, raising questions as to the nature of the host’s inflammatory state, microenvironmental factors, and epigenetic landscape, as discussed in a later section.

In recent decades, the incidence of CAC has been gradually declining [4]. This coincides with improvements in medications to control chronic inflammation in IBD despite mixed evidence supporting this link discussed below, better endoscopic detection of precursor lesions, and improved endoscopic resection techniques. Despite these positive developments, CRC rates remain higher in patients with IBD than the general population, and the increased risk applies even to those patients who enter remission.

A potential cancer chemopreventive effect of medications used to treat IBD has demonstrated mixed results [3]. The strongest data, as illustrated by meta-analysis, suggests that use of thiopurines (odds ratio: 0.55) and 5-aminosalicylic acid (odds ratio: 0.53) are associated with a lower risk of developing advanced colorectal neoplasia (high-grade dysplasia or carcinoma) [3]. The other mainstays of IBD treatment such as tumor necrosis factor-alpha inhibitors and corticosteroids, or other agents found to be chemopreventive for sporadic colorectal cancer such as nonsteroidal anti-inflammatory drugs, acetylsalicylic acid, folic acid, statins, or calcium supplements, were not associated with a lower risk of colorectal neoplasia in the setting of chronic colitis [3]. The reason for this paradox is unknown but suggest residual levels of inflammation after response to IBD treatment may nevertheless be sufficient to drive tumorigenesis. Thus, while controlling the underlying inflammation with medications is clearly important, regular endoscopic surveillance with removal of dysplastic precursor lesions continues to be predominantly responsible for reducing the incidence of colorectal neoplasia in IBD.

Like sporadic colorectal carcinogenesis, cancers in IBD arise from precursor dysplastic lesions. Although itself a benign transformation, dysplasia nevertheless confers a four-fold increased risk of cancer [4]. In the setting of colitis, dysplastic lesions progress from indefinite dysplasia to low- then high-grade dysplasia before becoming CAC. Importantly, CAC can develop from low-grade dysplasia (LGD) without apparently progressing through high-grade dysplasia (HGD) [5]. Dysplasia in IBD is often multifocal throughout the colon; the *field cancerization* effect of chronic inflammation on large swaths of epithelial cells leads to high rates of synchronous and metachronous dysplasia. Recovery from colitis involves the development of physiologic serrated or hyperplastic mucosal epithelium, so finding serrated epithelial changes in chronic colitis may be an innocent reflection of the healing process. More research is needed to better define the potential carcinogenic role of serrated lesions in colitis especially since they are often endoscopically invisible and detected as incidental findings on random biopsy [6, 7].

Because CACs are not usually encountered until individuals have had their colitis for at least eight years, endoscopic surveillance for CAC lesions typically begins at this time following IBD diagnosis [4]. Why it takes almost a decade of inflammation before most individuals develop colorectal neoplasia is not known. However, some patients develop CAC sooner than eight years of colitis, and ~13% of patients exhibit early cancer, found to have CAC at the time of their initial IBD diagnosis [8].

An important difference is that, unlike sCRC where precursor lesions are typically polypoid and therefore endoscopically visible, CAC poses a surveillance challenge with dysplastic lesions in colitis that are often flat and endoscopically invisible, necessitating the use of dyes during endoscopy for better visualization [9]. This has been a challenge to model in mice with commonly used azoxymethane/dextran sodium sulfate (AOM/DSS), which couples the mutagenic substance AOM with the inflammation-inducing detergent DSS, to model CAC. This rudimentary model is utilized for its ease of use, lack of genetic engineering or breeding, and speed, but may not fully reflect key aspects of CAC, such as *TP53* mutations resulting in flat lesions as compared to heterogeneous mutations induced by AOM, frequently in *APC*, that produce polypoid lesions characteristic of sCRC. Furthermore, there is a temporal discrepancy. CAC in humans is the result of years of preceding inflammation in the setting of IBD, whereas AOM/DSS results in rapid chemical mutagenesis. This highlights a need in the field to engineer more faithful mouse models of CAC.

Recently, a new mouse model of CAC genetically engineered mice to express the dominant negative TGFβ receptor II in CD4 and CD8 T cells (CD4-dnTGFβRII/AOM), and after challenge with a single dose of AOM, they formed macroscopically invisible flat adenocarcinoma lesions [10]. Although lesions were not sequenced, immunohistochemical studies on these lesions were non-reactive for p53, suggesting loss. Taken together, this highlights a promising advance in modeling CAC.

The mechanisms leading to invasive cancer are complex, but recent advances have improved our understanding of the genetic, epigenetic, and environmental factors involved. This Review focuses on the most significant advancements of the past five years.

## Field cancerization of nondysplastic IBD colon

CAC is thought to arise from the expansion of pro-tumorigenic clones that replace wild-type colorectal epithelium during chronic inflammation-induced field cancerization of the large intestine [11]. Somatic driver mutations arising from field cancerization have been observed in non-dysplastic inflamed colon years before CAC lesions are diagnosed [12]. Ulcerative colitis exhibits higher mutational burden, as much as 25-fold, compared to healthy colon [13]. It has been estimated that healthy colon crypts exhibited 40 somatic substitutions on average per year, whereas in the setting of IBD, non-dysplastic crypts showed 95 somatic substitutions on average per crypt per year of IBD duration [14, 15]. Thus, non-dysplastic crypts from the IBD colon are associated with a ~2.4-fold increase in mutation rate compared to those from normal colon.

A recent study suggests chronic inflammation in IBD accelerates existing, rather than unique, mutational processes in healthy colon crypts [14]. Analysis of specific mutational signatures found few differences between healthy and IBD crypts. Five out of seven IBD patients with past treatment of agents that disrupt purine synthesis, such as azathioprine and mercaptopurine exhibited a unique mutational signature. However, no unique mutational signature was linked to chronic inflammation itself, with a separate study finding the most frequent mutational signature in CAC to be one associated with aging [8].

Field cancerization induces both passenger and driver mutations that confer fitness against microenvironment stressors, including inadequate extracellular growth factor signaling, senescence, hypoxia, oxidative stress, and acidosis [16]. Individual point mutations serve as markers of clonality in phylogenetic studies. Whether a single mutation is temporally conserved across non-dysplastic, low-grade and high-grade dysplasia, and eventually carcinoma gives vital information on its origins and its significance towards cancer fitness [11, 12, 17, 18].

Multiple recent studies leveraged whole exome sequencing on laser micro-dissected, non-dysplastic bulk crypts isolated from inflamed regions in patients with ulcerative colitis (UC) for high-resolution profiling of the genomic landscape of field cancerization. These studies validate previous key observations in field cancerization preceding CAC. A study performed in the United Kingdom identified several TCGA hot spot mutations in well-known cancer driver genes such as *KRAS, BRAF, ERBB2, ERBB3, TP53*, and *FBXW7* in non-dysplastic mucosa, corroborating other reports of *TP53* and *KRAS* mutations in nondysplastic IBD colon, albeit at low frequency [14, 19, 20]. A similar study performed in Japan reproduced these findings, showing that *KRAS* mutations were enriched in non-dysplastic UC colon at approximately 14% (Table 1) [13].

**Table 1:** Top recurrent mutations between non-dysplastic UC and CAC.

Gene	Non-dysplastic IBD epithelium	Dysplastic IBD epithelium	CAC
*NFKBIZ*	30.1%	–	0%
*TRAF3IP2*	4.4%	–	1.0%
*PIGR*	20.8%	–	8.1%
*ZC3H12A*	13.7%	–	1.0%
*ARID1A*	24.0%	–	6.1%
*FBXW7*	9.8%	10%	9–11%
*APC*	1.6%	31%	14–20%
*RNF43*	0.6%	14%*	13.1%*
*KRAS*	13.7%	21%	27–31%
*BRAF*	<0.5%	7%^&^	4%^&^
*SMAD4*	0.5%	10%	12%
*TP53*	1.6%	52%	62–90%

Frequency of mutations in clones from non-dysplastic IBD colon, dysplastic IBD epithelium, and CAC. Note that mutations in the IL-17-NF-κB signaling pathway enriched in non-dysplastic IBD colon are poorly recapitulated in CAC suggesting they are negatively selected against during tumorigenesis. ^*^RNF43 mutations in dysplasia and CAC are mutually exclusive to *APC* mutations. ^&^*BRAF* mutations in dysplasia and CAC are mutually exclusive to *KRAS* mutations. – Data unpublished or unavailable.

A high ratio of non-synonymous to synonymous (dN/dS) mutations further identifies mutations under selection and therefore likely to be functionally relevant. Intriguingly, the UK study found most established driver genes did not demonstrate a dN/dS in the context of nondysplastic colon. Instead, they identified only four genes exhibiting a high non-synonymous to synonymous mutation ratio in non-dysplastic IBD, including *PIGR, ZC3H12A*, and two tumor suppressors, the SWI/SNF family chromatin remodeling gene *ARID1A* and the ubiquitin ligase complex gene *FBXW7* [14]. *PIGR* and *ZC3H12A* are inflammatory response genes which will be described more in-depth later in this section.

**Fig. 1:**
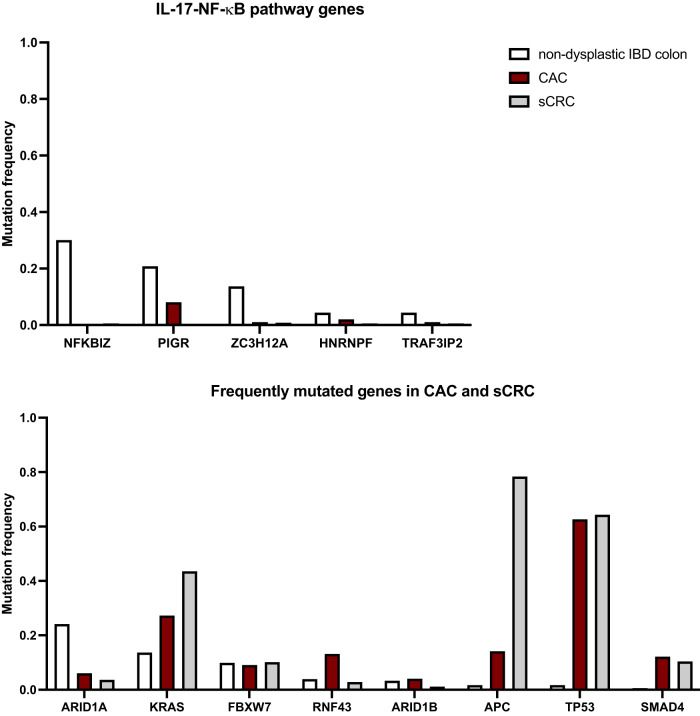
Top recurrent mutations between nondysplastic UC and CAC. Frequency of driver mutations in clones from ulcerative colitis nondysplastic colon, colitis-associated carcinoma (CAC), and sporadic colorectal carcinoma (sCRC) in **top** IL-17-NF-kB pathway genes and **bottom** frequently mutated established cancer drivers. Non-dysplastic UC most recurrently enriches for IL-17-NF-kB signaling pathway mutations, in addition to more well-recognized cancer drivers such as *ARID1A, FBXW7*, and *KRAS*. Intriguingly, these pathway mutations are virtually absent in CAC tumors, as well as sCRC. Conversely, the most recurrently mutated genes in CAC tumors are poorly represented in non-dysplastic UC except *KRAS*. Figure reproduced from Kakiuchi et al., 2019, with permission from Springer Nature. For more details, please refer to the original study.

*FBXWY* and *ARID1A* are mutated at similar, albeit relatively low, rates in sCRC and CAC (Fig. 1) [13, 21]. The role of *ARID1A* as a tumor suppressor in CRC was previously validated using genetically engineered mouse models, where *ARID1A* null mice exhibited increased spontaneous tumor formation in the large intestine and decreased overall survival following systemic treatment with the inflammatory stimulant Poly(I:C) [22]. In further support of SWI/SNF functional relevance, mutations in another complex member *ARID1B* are present at low frequency (~5%) in both nondysplastic UC and CAC [13]. *FXBW7* is a well-recognized E3 ubiquitin ligase component that recognizes pro-growth proteins for proteasomal degradation, most notably c-Myc, cyclin E, and mTOR complex members [23]. Low expression or loss of *FXBW7* correlates with increased cellular proliferation and decreased post-operative survival in CRC [24].

Recently, three independent groups performed unbiased whole exome sequencing on non-dysplastic IBD mucosa towards uncovering novel genetic insights that may have eluded previous targeted sequencing approaches. These different studies and cohorts highly corroborated each other and identified top recurrent mutations in *NFKBIZ* (30% of samples)*, PIGR* (21%)*, ARID1A* (14%)*, ZC3H12A* (14%), and *TRAF3IP2* (4%). These were previously undescribed in field cancerization within non-dysplastic mucosa preceding CAC as earlier studies were largely biased towards established cancer drivers [13, 14, 25].

Interestingly several recurrently mutated genes converge on the IL-17—NF-$\kappa$B signaling pathway (Table 1; Fig. 1) [25, 26]. The positive regulators of IL-17—NF-$\kappa$B signaling, which frequently exhibit truncating or other loss-of-function (LOF) mutations, are as follows. The top gene *NFKBIZ* is a positive feedback regulator induced by IL-17 activation of NF-$\kappa$B that further potentiates signaling [25]. *TRAF3IP2* is an adaptor for the IL-17 receptor which mediates activation of downstream NF-$\kappa$B signaling [25]. *PIGR* is a target of IL-17—NF-$\kappa$B signaling, which participates in the secretion of IgA for mucosal immunity [25].

These mutations are loss-of-function, frequently truncating mutations; the Japan study identified high dN/dS ratios for *PIGR, NFKBIZ, ZC3H12A*, the IL-17 receptor *IL17RA*, as well as the tumor suppressor *ARID1A*, suggesting functional selection in non-dysplastic IBD colon [25].

Conversely, *ZC3H12A* is a known suppressor of autoimmune disorders as an RNase that mediates mRNA degradation of *IL6* as well as *PIGR* and *NFKBIZ* transcripts [27]. Uniquely, 9/15 *ZC3H12A* mutations were hotspot, missense gain-of-function (GOF) mutations; these mutations specifically target serine residues within a phospho-degron motif within ZC3H12A in a way that renders it resistant to ubiquitination and proteasomal degradation [25]. Subsequently, GOF ZC3H12A increases the degradation of *PIGR* and *NFKBIZ* mRNA. Thus, all mutations weaken the IL-17—NF-$\kappa$B signaling pathway, possibly as a negative feedback loop to suppression inflammatory signaling.

These mutations were observed to confer a fitness advantage. Colon organoids harboring genetic deletions of *NFKBIZ* mimicking LOF mutations in non-dysplastic IBD colon were more protected against IL-17A toxicity compared to WT controls [25]. Similarly, organoids bearing the *ZC3H12A*^*D437Y*^ GOF hotspot mutation also exhibited greater fitness in response to IL-17A [25]. However, this is incongruent with human data, as clinical trials utilizing brodalumab, an anti-IL-17-receptor monoclonal antibody for upstream targeting of this pathway, paradoxically worsened Crohn’s disease in patients and resulted in early trial termination [28].

Perhaps most surprisingly, these recurrent IL-17—NF-$\kappa$B pathway mutations observed in non-dysplastic IBD mucosa are poorly represented in primary CAC and CAC-derived organoids [13, 25]. In an analysis comparing 183 independent non-dysplastic UC colons, 99 CACs, and 356 sporadic CRCs, *NFKBIZ* mutations were detected in 30% of non-dysplastic colons but less than 5% of CACs and sCRCs (Table 1, Fig. 1) [13]. Similar trends were observed for *PIGR, ZC3H12A*, and *ARID1A* mutations, present in greater than 20% of non-dysplastic UCs but less than 10% of CACs (Table 1, Fig. 1) [13].

The absence of IL-17—NF-$\kappa$B pathway mutations in CAC raises the possibility that positive selection during chronic inflammation may switch to negative selection at some point during neoplastic transformation. Indeed, mice with *NFKBIZ* conditionally deleted in the colon were protected against tumors induced by AOM/DSS, rather than advancing tumor burden [13]. These recent data provocatively suggest that the most highly recurrent mutations incurred because of field cancerization in UC may be functionally irrelevant to CAC tumorigenesis. It is currently unclear whether these mutations remain enriched in, or are selected out, in low- or high-grade dysplasia, a promising space for future exploration that could provide further understanding of how selective pressures transition over time. Furthermore, future studies should further elucidate the molecular basis of this novel *NFKBIZ* tumor suppression in the setting of CAC.

A recently published study demonstrated a similar observation in esophageal epithelium, where mutations in *NOTCH1* drive clonal expansion in normal esophagus but are less prevalent in esophageal carcinomas where they are found to paradoxically inhibit tumor growth [29]. These observations differ significantly from those seen in pre-malignant field cancerization patterns in skin and hematopoietic cells, where most acquired mutations are present in the resulting cancers [30–34]. These advances further serve to highlight the unique genetics of field cancerization preceding CAC compared to sCRC.

## Aneuploidy in IBD nondysplastic colon, dysplasia, and CAC

For decades, it has been known that aneuploidy is an early event in CAC carcinogenesis. Serial biopsies in patients with UC demonstrated aneuploidy spread over a large surface area of inflamed but otherwise non-dysplastic epithelium that predicts future dysplasia [35]. Aneuploidy events—including copy number variation (CNV), retrotranspositions, and loss-of-heterozygosity of chromosomes or chromosomal arms—are observed at three times the frequency in IBD colon as compared to normal colon controls [14].

**Fig. 2:**
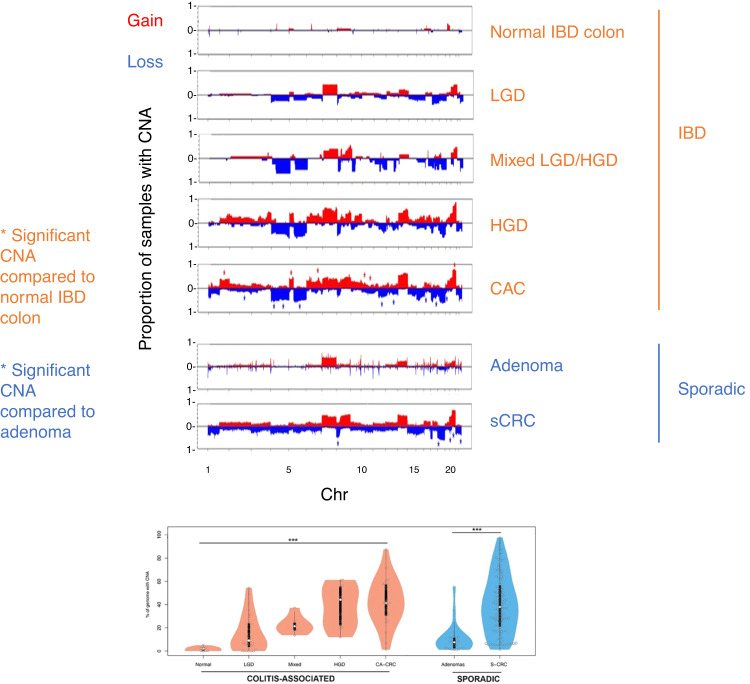
Aneuploidy is an early finding in colitis-associated dysplasia. A visualization of genome-wide copy number alterations in normal IBD colon, low-grade dysplasia IBD colon (LGD), mixed-grade IBD colon (LGD/HGD), high-grade dysplasia IBD colon (HGD), and colitis-associated carcinoma (CAC). Sporadic CRC tumors used for comparison are microsatellite stable. Blue or red bars indicate statistically significant arm losses or gains, respectively. Figure reproduced from Baker et al., 2019, reproduced under an open-access Creative Commons CC By 4.0 license. For more details, please refer to the original study.

Aneuploidy is observed in as much as 41% of non-dysplastic lesions of patients from IBD and continues to accrue until it becomes a virtually obligatory finding in CAC that is frequently associated with p53 loss (Fig. 2) [13, 36]. In one study, it was present in 14 out of 15 high-grade dysplasia samples from 9 out of 10 patients with IBD [37]. While the number of aneuploidy events in non-dysplastic or low-grade dysplasia is low, the transition from low-grade dysplasia to high-grade dysplasia is associated with the greatest increase in aneuploidy events [21]. In a small study of 37 samples, aneuploidy in flat low-grade dysplasia was associated with significantly increased subsequent detection of high-grade dysplasia or CRC (hazard ratio 5.3), compared with the same lesions without aneuploidy [37].

Interestingly, recent studies using laser capture microdissection to sample multiple colon crypts from the same IBD patient reveal that broad increases in aneuploidy in IBD colon are contributed by a few select clones, whereas most of the inflamed IBD colon did not exhibit any structural variation [14]. This finding agrees with recent models suggesting that extensive copy number variation observed in CRC is episodic, arising from a few bursts akin to the “Big Bang” model as discussed later, rather than a gradual continuous accumulation over time [38]. The number of aneuploidy events was not significantly correlated with IBD duration possibly for this reason [14].

CACs exhibit only few regions of unique allelic imbalance compared to sCRC [39]. A single region on chromosome arm 5p is selectively gained in CAC over sCRC, notably containing the cytokine receptors *OSMR* and *LIFR* [39]. CAC also shows hypomethylation of *OSMR* with strong overexpression, as a complement to amplification of its locus on chromosome arm 5p in CAC [39]. At the gene level, the tumor suppressors *CDKN2B* and *SMARCA2* exhibited allelic losses in CAC, whereas the transcription factors *FOXA1* and *HNF4A* exhibited allelic gains in CAC [39]. The proto-oncogene and regulator of cell proliferation *MYC* was found to be amplified in ~20% of advanced CACs but not in IBD low or high-grade dysplasia, suggesting a late event [8].

## Overview of the somatic mutation landscape in IBD dysplasia and CAC

*TP53, KRAS*, and *SMAD4*, which are frequently mutated in sCRC, are among the most recurrent gene mutations in CAC (Fig. 1) [40, 41]. Alternatively, *APC* mutations, which are observed in 81% of sporadic microsatellite stable (MSS) CRC cases by TCGA analyses, are detected in only 11.1% [42], 13% [41], or 22% [40] of CACs in independent cohort studies; they are present in IBD dysplasia as well at similarly low levels (~31% in a low powered study) (Table 1) [8]. *APC* encodes an essential member of the β-catenin destruction complex, which critically attenuates β-catenin nuclear localization and association with TCF family transcription factors to promote Wnt signaling [43]. As such, immunohistochemical staining shows decreased nuclear $\beta$-catenin in CAC compared to sCRC [21]. The disparity of *APC* mutations suggests the presence of an alternative mechanism of tumor initiation in CAC. *APC* mutations often occur late in CAC progression, indicating it may be dispensable for tumor initiation for this unique subset of CRC [39].

**Fig. 3:**
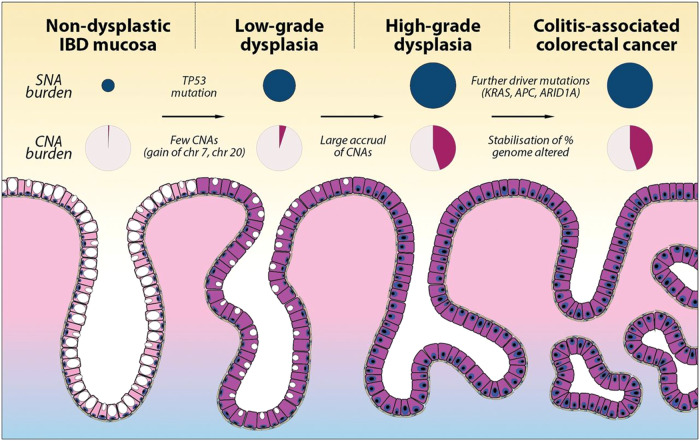
Overview of genomic alterations and their sequence in CAC. In contrast to the classic Vogelgram model of sCRC progression, p53 loss is an early event in CAC rather than late. *APC* mutations on the other hand, are observed to occur after p53 loss rather than before, as classically observed in sCRC. Copy number alteration and aneuploidy are substantial in both CAC and sCRC, with similar regions experiencing gains and losses in both. Figure reproduced from Baker et al., 2019, reproduced under an open-access Creative Commons CC By 4.0 license. For more details, please refer to the original study.

The appearance of *TP53* missense mutations early during CAC poses one alternative mechanism of tumorigenesis compared to sCRC, where they are thought to occur late (Table 1, Fig. 3) [44–48]. *TP53* mutations are by far the most prevalent in IBD dysplasia, observed in approximately half of cases. As is observed in other cancers, the majority of p53 mutations are gain of function (GOF) missense mutations within the DNA binding domain [47]. The spectrum of p53 GOF mutations between CAC and sCRC are largely similar, although some exceptions have been proposed. One study noted a paucity of R273, R248, G245, and R175 hotspot mutations, frequently observed in almost all cancers, in CAC compared to sCRC [41]. Conversely, CAC may exhibit more uncommon missense mutations at R158, H179, and R342 compared to sCRC, where they are rarely seen [41]. However, drawing conclusions from such comparisons is limited by power, and may merit a future meta-analysis for more concrete insight.

Some mutations are contingent on inflammation being present in the microenvironment to affect tumorigenesis [18]. p53, whose hallmark feature is the ability to coordinate diverse transcriptional programs depending on tissue identity and individual stressors, interplays with inflammation in CAC as confirmed in various mouse studies [49]. For instance, deletion of *Trp53* (p53) in murine colorectal epithelium accelerates pre-malignant stem cell replacement in the setting of experimentally induced colitis but not under uninflamed physiologic conditions. This may explain the selection of p53 mutations early in CAC but late in sCRC. The phenomenon where inflammation creates a permissive environment for driver mutations to form tumors is also observed in pancreatic cancer, where local tissue injury results in inflammation that cooperates with *KRAS* mutations for transformation [50].

Several genetically engineered mouse models (GEMMs) suggest these missense gain-of-function (GOF) *TP53* mutations sustain inflammatory signaling in the setting of colitis. In one study, a GEMM modeling the R172H mutation in mice—corresponding to the R175H hotspot mutation observed in human cancers—heightened NF-$\kappa$B activation in response to TNF-$\alpha$ and increased tumor burden by DSS as a novel GOF activity [41, 51]. A separate GEMM modeling the human *TP53* R248Q hotspot mutation similarly discovered GOF activity sustaining JAK2/STAT3 signaling activity [52]. Genetic ablation of the mutant p53 allele from *Trp53*^*R248Q/−*^ to *Trp53*^*−/−*^ diminished tumor formation, proliferation, and invasion in an AOM/DSS mouse model of CAC [52]. The ablation of the R248Q mutant p53 also decreased phosphorylation and inflammatory signaling JAK2 and STAT3 [52]. The pro-tumorigenic roles of STAT3 are well-described in CRC [53]. Interestingly, the R248Q mutant failed to demonstrate increased NF-$\kappa$B signaling as well as nuclear retention of the complex observed in the R175H mutant, suggesting p53 mutant-specific neomorphic activity.

In addition to *TP53*, studies have also demonstrated a role for *SMAD4* in regulating inflammation. Pathway analyses of non-dysplastic colonic epithelium in a GEMM of *SMAD4* loss shows upregulation of multiple inflammatory signaling pathways, including IL-6/STAT3 and NF-$\kappa$B [54]. Studies show *SMAD4* mutations to first emerge in IBD dysplasia (10%) and levels persist stably to CAC (Table 1).

The frequency of *KRAS* mutations in CAC varies between studies, estimated to be either equal to, or slightly lower than, the frequency observed in sCRC [13, 40, 41]. The frequency of *KRAS* mutations rise continuously from non-dysplastic IBD colon to CAC, and are observed to be mutually exclusive with mutations in *BRAF* (Table 1). Meta-analyses suggest flat or invisible lesions in patients without IBD exhibit less *KRAS* (OR 0.42, CI 0.24-0.72) and *APC* (OR 0.3, CI 0.19-0.46) mutations compared with polypoid lesions, but with an increased frequency of *BRAF* mutations (OR 2.2, CI 1.01-4.81) [55].

**Fig. 4:**
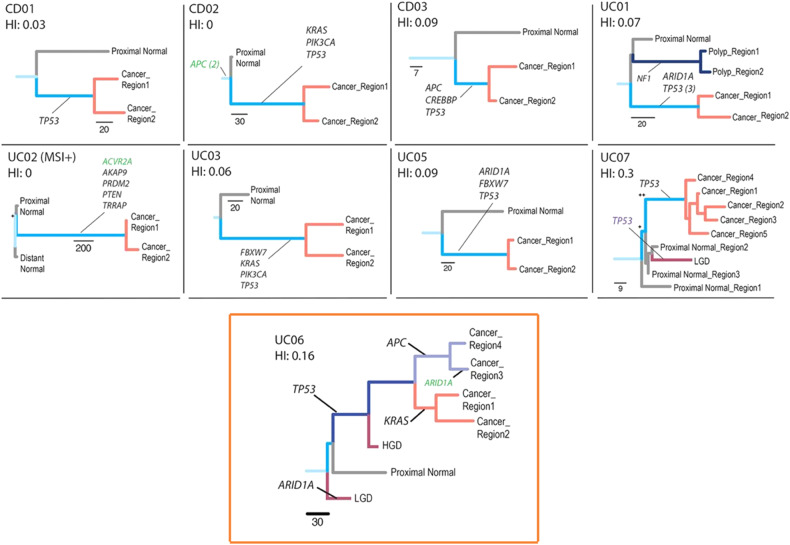
Single nucleotide alteration phylogenic analysis of CAC. Phylogenetic trees constructed from eight independent ulcerative colitis (UC) or Crohn disease patients (CD). Driver mutations, annotated, appear early and simultaneously in phylogenetic history. Top, 8 cases showing simultaneous truncal emergence of multiple mutations in a single clone. Bottom, 1 case showing sequential truncal emergence of multiple mutations accumulating over genetically distinct clones (UC06, boxed in orange). Figure reproduced from Baker et al., 2019, reproduced under an open-access Creative Commons CC By 4.0 license. For more details, please refer to the original study.

In contrast to the sequential evolution model of CAC, where driver mutations are accumulated over time, a competing recent “Big Bang” model of CRC was proposed in which the accelerated mutagenesis in the setting of colon inflammation results in abrupt tumor-initiating mutations arising simultaneously in a single clone, rather than a chronic accumulation guided by selective pressures from the environment [56]. A recent study performed by Baker et al. supports this latter model. The authors sampled multiple sites within CAC as well as surrounding dysplastic and nondysplastic colon to construct phylogenetic trees. In 8 out of 9 cases, they found early presence of conventional driver mutations, such as *KRAS*, and *TP53*, originated clonally, meaning multiple mutations spontaneously emerged within a single clone as posited in the “Big Bang” model (Fig. 4) [21]. Only one out of 9 cases showed mutations accumulating individually across several genetically distinct clones as per the sequential evolution model (Fig. 4, see orange box). While data exists to support both models, high-power and high-resolution phylogenetic studies that sample low- and high-grade dysplasia and normal mucosa around CAC within a single patient will continue to shed light on the evolutionary dynamics of CAC.

## Transcriptomics and epigenetics

As discussed earlier, a subset of CAC associated with primary sclerosing cholangitis (PSC), a condition where the bile duct becomes inflamed and subsequently scarred and stenotic, is particularly tumorigenic despite clinically milder inflammation in the colon [19]. However, PSC-CAC and non-PSC CAC exhibit similar genomic alterations, including copy number alterations and mutations. In a cohort of 19 PSC-CAC patients, the largest assembled for mutational analyses to date, *TP53* remains the most frequently mutated gene (~68%) while less frequently exhibiting mutations in *APC* (5%) and *KRAS* (5%) compared to non-PSC-CAC [19]. These observations suggest the possibility of epigenetic drivers underlying PSC-CAC and highlight a need for epigenetic and transcriptomic characterization in search of alternative, non-mutated drivers [19].

**Fig. 5:**
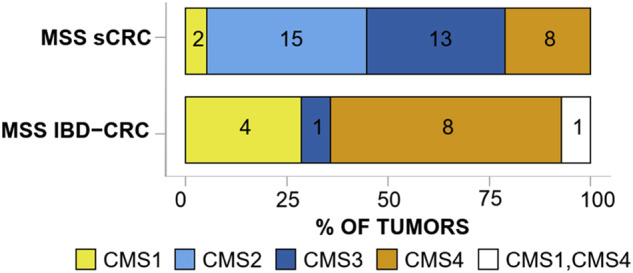
The consensus molecular subtypes of CAC. Bulk tumor RNA-seq of independent microsatellite stable sporadic CRC (MSS sCRC) and microsatellite stable colitis-associated CRC (MSS IBD-CRC) tumors were binned into the consensus molecular subtypes of CRC. This initial study suggests CAC may most transcriptionally aligned with the CMS4 subtype, characterized by stromal, CD4 + T cell, and monocyte involvement. Figure from Rajamaki et al., 2021, reproduced under an open-access Creative Commons BY-NC-ND 4.0 license. For more details, please refer to the original study.

Bulk tissue RNA-seq of sCRC and CAC indicate divergent transcriptomes. In keeping with the lower frequency of *APC* mutations and decreased nuclear $\beta$-catenin staining, enrichment of a Wnt/TCF signaling gene signature is also lower in CAC [39]. Notably, increased gene signatures associated with epithelial-mesenchymal-transition were found in CAC compared to sCRC [39]. These results validate consensus molecular subtype (CMS) analyses (Fig. 5) [57, 58]. MSS sCRC mostly cluster in the Wnt-driven CMS2 subtype and MSI sCRC in the CD8+ cytotoxic T cell-driven CMS1 subtype [39, 57, 58]. When it comes to CAC, bulk RNA-seq best match the CMS4 subtype of CRC characterized by strong stromal, CD4 + T cell, and monocyte involvement as well as NOTCH1 signaling [59]. CMS4 tumors are characterized by an enrichment of cancer-associated fibroblasts, which are also associated with poor prognosis in rectal cancer and resistance to neoadjuvant radiation therapy, which by current guidelines is considered for rectal but not colon cancer [60–63]. Specifically, the release of cytokine IL-1 from rectal cancer cells predisposed these cancer-associated fibroblasts towards p53-mediated senescence because of IL-1-mediated oxidative damage [60]. This in turn led cancer-associated fibroblasts to become resistant to primary tumor shrinkage by radiation therapy, a phenotype that is reversed with IL-1 blockade [60]. Similarly, chemotherapy has been shown to increase the number of cancer-associated fibroblasts in CRC, which in turn release the pro-inflammatory cytokine IL-17A [64]. Another study has shown myofibroblasts in CAC to produce pro-inflammatory and pro-CAC cytokines in an MYD88-dependent manner [65].

The study of cancer-associated fibroblasts in tumorigenesis and tumor progression is vast and covered in several excellent Reviews, only a fraction of which is covered here [66–68]. Altogether, the intriguing trend of CMS4 enrichment among CACs await reproducibility using larger cohorts.

Inflammation shapes the epigenetic landscape through de novo formation of enhancer, super-enhancer, and open chromatin loci [50, 69–71]. Few studies have assessed the methylation or acetylation landscapes of CAC on a global scale. Nascent studies show the level of methylation between CAC and MSS sCRC genome-wide remains largely unchanged, and CAC shows lower global methylation levels compared to non-neoplastic IBD colon [39]. However, an exception may be *IDH1* mutant CACs.

*IDH1* mutations in CAC (7% in both IBD dysplasia and CAC; not reported in non-dysplastic IBD colon) follow hotspots observed in other cancers, such as gliomas, that result in accumulation of the 2-hydroxyglutarate oncometabolite (2-HG) which results in a hypermethylation phenotype [8, 72–74]. These have been observed to displace CTCF from their binding sites, proteins that enforce “gene neighborhoods” and regulate gene expression by ensuring distal enhancers are insulated to only activate genes within their neighborhood, a guardian role known to be tumor suppressive. In glioma and gastrointestinal stromal tumors (GISTs), 2-HG displacement of CTCF allows enhancers once insulated from nearby proto-oncogenes to activate their expression to promote tumor growth and progression [75, 76]. Given tissue-specific enhancer and CTCF chromatin landscapes as well as cancer dependency genes, the epigenetic consequences of *IDH1* mutation in CAC and its interplay with inflammation will be an exciting area of future study [10, 77].

As discussed earlier, mutations in SWI/SNF chromatin remodeling members such as *ARID1A* and *ARID1B* are observed in CRC. However, the effects of such mutations on the enhancer and accessible chromatin landscape of CAC, and whether it interplays with inflammatory transcription factors, is poorly understood. Detailed open chromatin and enhancer mapping of CAC in comparison to sCRC may provide further novel insight into the epigenetic facets of pathogenesis.

## Microbiome

Crohn’s disease (CD) is associated with polymorphisms in *NOD2* (also known as *NLRC2*), an innate sensor of bacterial infection for immune response [78–80]. Another member of the same family of proteins, *NOD3* (also known as *NLRC3*), is observed to be silenced in CRC compared to healthy colon [81, 82]. Following AOM/DSS challenge, *Nlrc3*^*−/−*^ mice exhibited significantly higher colitis severity as well as CAC burden, with the majority of mice exhibiting features of adenocarcinoma whereas WT mice exhibited none [81]. Mechanistic studies in murine large intestinal organoids found loss of *Nlrc3* promoted mechanistic target of rapamycin (mTOR) and phosphoinositide 3-kinase (PI3K) signaling in response to toll-like receptor activation. Taken together, this data implicates *NOD3* as a regulator of colitis and CAC in addition to the more well-characterized *NOD2* [81].

Certain species of colonic bacteria have been linked to CRC. *Fusobacterium nucleatum, Escherichia coli, Bacteroides fragilis, Enterococcus faecalis, Streptococcus gallolyticus, and Peptostreptococcus* species are among the microbes most-documented as upregulated in CRC [83]. Some species are observed to be more prevalent in the setting of IBD.

*Escherichia coli* species harboring a polypeptide-non-ribosomal peptide synthase operon (*pks*) produce colibactin, a DNA alkylating and double-stranded break-inducing genotoxin. These species are increased in the colonic mucosa of both IBD and CRC patients, with an estimated 20% prevalence in healthy individuals and 40% in IBD patients [84, 85]. DNA, in particular adenine, is alkylated by this metabolite [86, 87]. In support, in vitro models indicate colibactin induces a DNA-damage signature present in AT-rich hexamer motifs [88]. A pan-cancer mutation signature analysis identified an isolated enrichment in primary CRC where these microbes are found, highlighting the disease relevance of this genotoxic microbial metabolite [88]. Indeed, a separate study identified the presence of colibactin-producing *E. coli* species in early adenomas from familial adenomatous polyposis patients [89].

The pro-tumorigenic role of *pks* + *E. coli* has been demonstrated using many independent mouse models of CAC. Inoculation of *pks* + *E. coli* into colitis-prone *Il10*^*−/−*^ mice resulted in greater intestinal tumor burden and invasion, compared to *pks- E. coli* [90]. Interestingly, the level of intestinal inflammation did not differ between the two species [90]. In a separate *Apc*^*min*^*/Il-10*^*−/−*^ mouse model, tumor necrosis factor-alpha (TNF-α) neutralizing antibodies attenuated colibactin-producing *E. coli-induced* colitis and CRC development, highlighting the significance of inflammatory signaling in tumorigenesis [91]. Intriguingly, TNF-α blockade decreased bacterial expression of *pks+* islands without affecting colonization of *pks* + *E. coli*, suggesting microbial transcriptional plasticity [91].

In metagenomic studies, *B. fragilis* species were decreased in IBD patients, but increased overall in primary CRC compared to normal healthy controls [92, 93]. Like *pks* + *E. coli*, enterotoxigenic *Bacteroides fragilis* (ETBF) secretes a toxin with multiple reported effects on *Apc*^*min*^ colon epithelial cells, including an IL-17 inflammatory cascade with induction of c-Myc, STAT3, and NF-κB signaling [94, 95]. In an AOM-induced model of colitis, *pks* + *E. coli* inoculated mice alone did not increase tumor burden, but co-inoculation of *pks* + *E. coli* with ETBF significantly increased tumor burden in this disease model with increased expression of IL-17 and downstream inflammatory signaling [89].

**Fig. 6:**
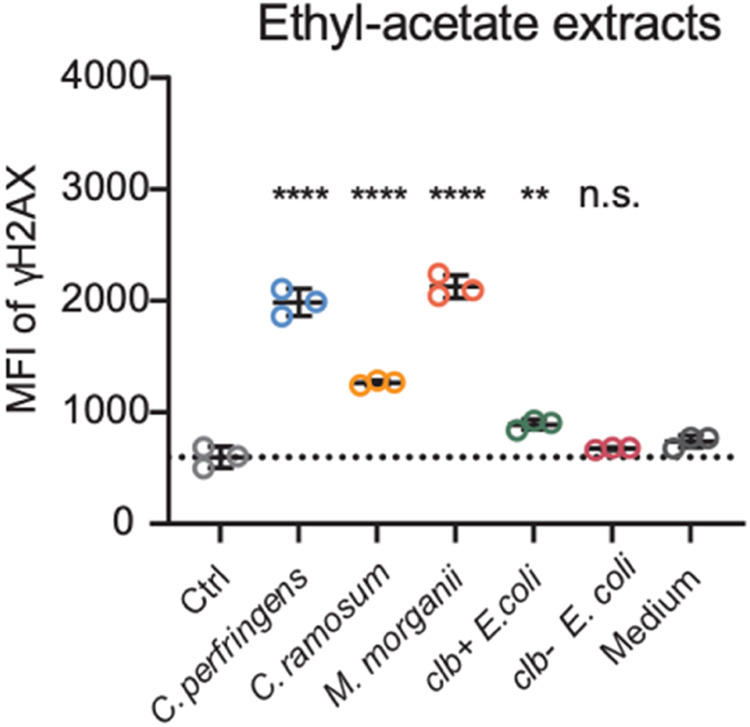
Genotoxic metabolites produced by the gut microbiome in IBD. Several *Clostridium* and *E. coli* species, as well as the recently characterized *M. morganii* produce novel genotoxic metabolites that appear more potent than those from colibactin producing *E. coli*. Mean fluorescent intensity (MFI) of the double-stranded DNA break marker γH2AX in HeLa cells treated with metabolite extracts from indicated bacterial strains, including the genotoxin colibactin (clb) producing and non-producing strains of *E. coli*. Figure from Cao et al., 2022, reproduced with permission from *Science*. For more details, please refer to the original study.

The full repertoire of genotoxic organisms and metabolites from the gut microbiome remains incompletely profiled. A recent proof-of-principle study screened more than one hundred gut commensal bacteria from IBD patients and identified colibactin-independent mechanisms of DNA damage (Fig. 6) [96]. *Morganella morganii*, enriched in both IBD and CRC patients, produced a novel class of genotoxins called indolimines via bacterial aspartate aminotransferase (*aat*) that increased tumor burden in a mouse model [96].

In addition, metagenomic studies also identify increased *Fusobacterium* species in IBD over healthy colon, a trend which persists into both primary CRC and distant metastases [92, 93, 97]. *Fusobacterium* species were conserved within CRC patient-derived xenograft (PDX) models, and treatment with metronidazole decreased both *Fusobacterium* load and PDX growth but failed to reduce xenograft growth of sterile CRC cell cultures, suggesting that on-target effect of antibiotics may slightly curtail CRC growth [98].

## Niche remodeling

Innate lymphoid cells (ILCs) are tissue-resident innate lymphocytes present at the intestinal mucosal barrier that regulate host-microbe interactions. Group 3 ILCs (ILC3s), which respond to microbiota in the intestines via the release of IL-17 and IL-22, were recently found using single-cell RNA-sequencing to be profoundly different in patients with colitis and CAC, with decreased population, as well as altered cellular plasticity and T cell interactions compared to controls [99]. This is corroborated by numerous past studies suggesting decreased functionality of intestinal ILC3s in patients with IBD.

Specifically, the authors found that ILC3 and T-cell interaction depends on major histocompatibility complex II (MHC-II) for microbiota-dependent immunity. This interaction was found to be critical for tumor growth, as deletion of MHC-II on ILC3s specifically significantly increased adenoma size and load in an AOM/DSS-induced model of CAC compared to WT mice [99]. Furthermore, AOM/DSS-induced CACs in ILC3-deleted mice exhibited decreased response to anti-PD-1 immunotherapy compared to WT mice, which exhibited a more than 90% reduction in tumor growth compared to controls, suggesting ILC3s critically influence response to current immunotherapies [99].

UC also exhibits expansion of a stromal compartment with pro-inflammatory and lymphoid signatures rarely detected in healthy colon. The cells responsible for these signatures secrete LIGHT protein and interleukin 6 (IL-6) upstream of STAT3 to modulate the immune response and format tertiary lymphoid structures in the setting of chronic inflammation [100]. This stromal compartment also expands in mice following treatment with DSS [100]. A separate study similarly identified a population of inflammation-associated fibroblasts unique to IBD colon, expressing genes associated with cancer, colitis, and fibrosis, including interleukin-11, also upstream of STAT3 [101]. Stromal activation of STAT3 via IL-6 is linked to both CRC and IBD. The pro-tumorigenic role of STAT3 in CRC and other cancers is well-established [102, 103]. A study of cancer-associated fibroblasts found increased phosphorylation of STAT3 to correlate with decreased overall survival in sCRC, with implications for CAC [104]. In a mouse model, expression of a constitutively active STAT3 mutant (STAT3C) within mouse colon fibroblast increased tumor burden in response to AOM/DSS [104]. Taken together, these data highlight microenvironment remodeling to further activate STAT3 signaling in epithelial cells towards cancer progression.

## Conclusion and future directions

Our understanding of how chronic inflammation and its consequent molecular mechanisms underlying CAC tumorigenesis continues to advance. Recent high-resolution crypt-sequencing studies allowed more sophisticated observation of genetic and cell population changes in the cancerized field. Furthermore, burgeoning transcriptional and epigenetic analyses between CAC and sCRC suggest a greater divergence than once expected. It is hoped that advances in genetic, epigenetic, immunologic, and microbiological molecular mechanisms can be leveraged to identify novel target pathways for the prevention and management of CAC.

Several broad, outstanding questions regarding field cancerization remain. A substantial minority of UC patients develop CAC after less than eight years following diagnosis and, conversely, the vast majority of UC patients never develop cancer, a disparity that needs to be framed in the context of both genetics and environment.

Clinical epidemiologic data suggests not all inflammation appears to confer the same risk for CAC. Why does colonic inflammation result in increased cancer risk but isolated proctitis does not; is this simply a reflection of the reduced area of mucosa at risk? Why does the presence of primary sclerosing cholangitis (PSC) in someone with IBD colitis confer some of the highest risk of CAC even if colitis is minimal, whereas isolated PSC confers no additional risk? A more granular profiling of immune cell populations and inflammatory cytokines may be needed to explain these subtype paradoxes of the CAC spectrum.

Perhaps most significantly, recent studies suggest shifting selective pressures that erase clones selected for during non-dysplastic colitis over the course of CAC tumorigenesis. Why do UC field cancerization mutations in the IL-17/NF-κB pathway “disappear” upon CAC formation, as opposed to mutations in other normal tissue epithelia upon aging, where they persist into their respective cancers? Do these pathways represent a previously unrecognized tumor suppressor function or are they simply outcompeted? Mechanistic experiments will provide further insight into this observation, which continues to be shaped by the continuingly increasing granularity in terms of temporality and histological context (non-dysplastic versus dysplastic versus neoplastic) in which transcriptomic, genomic, epigenomic, and metagenomic characterizations are being performed.

Lastly, greater efforts should be directed towards the generation of a mouse model that more faithfully recapitulates CAC. The AOM/DSS model, while fast, simple, and cost-effective, results in genetic heterogeneity as it is a chemical model. Lesions are also polypoid and may not reflect the pathology of human disease, which are flat lesions. Genetically engineered mouse models of p53 loss in colon may be a cleaner approach, in the setting of genetically (CD4-dnTGFβRII) or chemically (DSS) induced inflammation may result in a more authentic model and should be investigated. A faithful genetically engineered mouse model (GEMM) for CAC will permit high-resolution single-cell profiling of genetic, transcriptomic, and epigenomic events for cells throughout the sequential spectrum of timing and histopathology.
